# A gene expression signature in developing Purkinje cells predicts autism and intellectual disability co-morbidity status

**DOI:** 10.1038/s41598-018-37284-1

**Published:** 2019-01-24

**Authors:** Harry Clifford, Anna Dulneva, Chris P. Ponting, Wilfried Haerty, Esther B. E. Becker

**Affiliations:** 1 MRC Functional Genomics Unit, Department of Physiology, Anatomy and Genetics, University of Oxford, Oxford, OX1 3PT United Kingdom; 2 Present Address: Earlham Institute, Norwich Research Park, Norwich, NR4 7UG United Kingdom

## Abstract

Autism spectrum disorder (ASD) is a complex neurodevelopmental disease whose underpinning molecular mechanisms and neural substrates are subject to intense scrutiny. Interestingly, the cerebellum has emerged as one of the key brain regions affected in ASD. However, the genetic and molecular mechanisms that link the cerebellum to ASD, particularly during development, remain poorly understood. To gain insight into the genetic and molecular mechanisms that might link the cerebellum to ASD, we analysed the transcriptome dynamics of a developing cell population highly enriched for Purkinje cells of the mouse cerebellum across multiple timepoints. We identified a single cluster of genes whose expression is positively correlated with development and which is enriched for genes associated with ASD. This ASD-associated gene cluster was specific to developing Purkinje cells and not detected in the mouse neocortex during the same developmental period, in which we identified a distinct temporally regulated ASD gene module. Furthermore, the composition of ASD risk genes within the two distinct clusters was significantly different in their association with intellectual disability (ID), consistent with the existence of genetically and spatiotemporally distinct endophenotypes of ASD. Together, our findings define a specific cluster of ASD genes that is enriched in developing PCs and predicts co-morbidity status.

## Introduction

Autism spectrum disorder (ASD) is a highly prevalent, complex group of neurodevelopmental diseases defined by deficits in social cognition and communication as well as restricted interests and repetitive behaviours. Beyond these core features, ASD is often associated with variable co-morbid conditions including a low nonverbal intelligence quotient, motor deficits and epilepsy^1^. ASD is highly heritable, and recent advances in genomic technology have led to the identification of several hundred genetic variants associated with ASD^1^. This considerable genetic heterogeneity of ASD combined with its broad clinical phenotype present a major challenge to our understanding of the underlying disease pathophysiology. The primary molecular mechanisms and also the neural substrates that cause ASD remain largely to be elucidated.

Interestingly, the cerebellum has emerged as one of the key brain regions affected in autism^2,3^. Imaging meta-analysis has revealed a significant reduction of distinct grey matter areas in the cerebellum in ASD^4^, whose degree predicts the severity of core autism symptoms^5^. In particular, Purkinje cells (PCs), which constitute the sole output neurons of the cerebellar cortex, are reduced in number and density in ASD^1,6^. Moreover, a critical role for PCs in autism has been demonstrated in PC-specific conditional mouse models lacking the ASD-associated genes *Tsc1*, *Tsc2* and *Shank2*^7–9^. Together, these and other findings suggest that PC dysfunction during a critical developmental period may contribute to ASD^3,10^. Remarkably, developmental injury to the human cerebellum constitutes the largest single non-heritable risk of developing ASD^3^. However, our understanding of the genetic and molecular mechanisms that underpin developmental PC dysfunction and might trigger ASD remains poor.

To gain a better understanding of the genes expressed during PC development and their potential association with ASD, we profiled the transcriptome of developing mouse PCs by deep sequencing across multiple developmental time points. We identified a single cluster of genes whose expression is positively regulated over PC development and that is enriched for genes associated with ASD. This ASD-associated gene cluster was specific to developing PCs and was not associated with the mouse neocortex during the same developmental period, in which we identified a second temporally regulated ASD gene module. Strikingly, we recognized a significant difference in the composition of the ASD risk genes underlying the clusters in the two brain regions that relates to their association with intellectual disability (ID).

## Results

Increasing evidence points to a role for PC dysfunction during a critical developmental period in the causation of ASD^3,10^. The human cerebellum develops over a protracted period of time, ranging from 4 weeks of gestational age until 20 months of postnatal age^11,12^. The third trimester of pregnancy is characterized by a highly dynamic period for cerebellar development^13^, during which time the cerebellum is extremely vulnerable to insults that are strongly associated with autism^3,14^.

**Figure 1:**
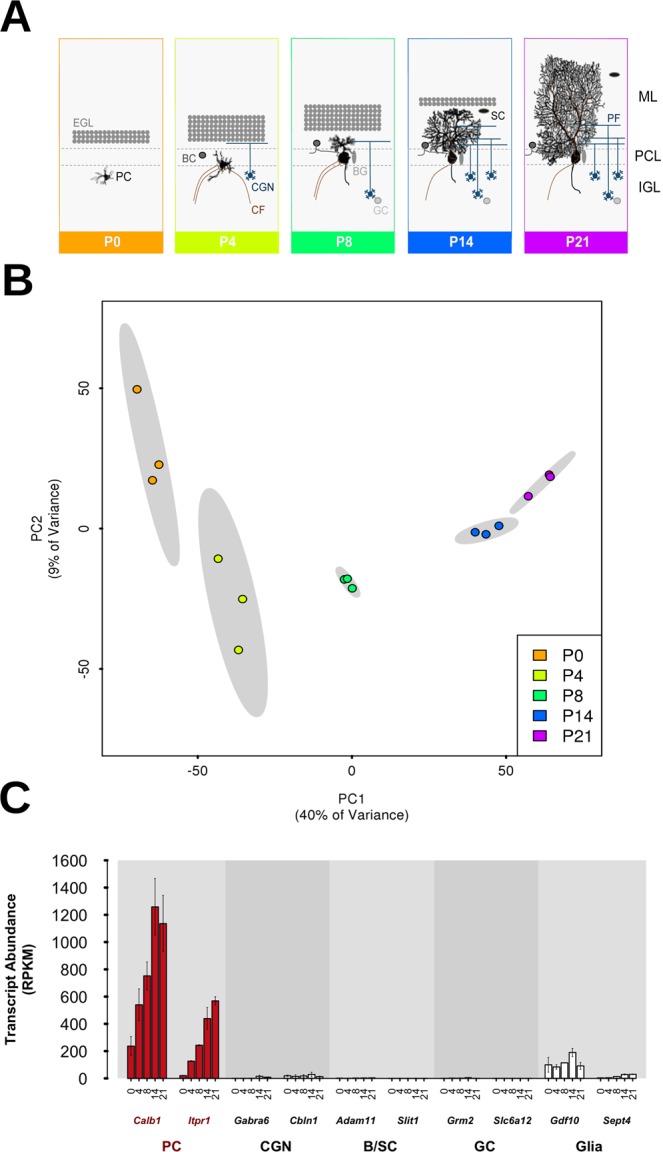
Gene expression data cluster according to developmental time point and confirm successful Purkinje cell (PC) capture. (**A**) Schematic diagram of mouse PC development from postnatal day 0 (P0) until P21. This developmental period is characterized by extensive morphological changes and the formation of synaptic connections with climbing fibers (CF), Basket cells (BC) and parallel fibers (PF) of cerebellar granule neurons (CGN). BG: Bergmann glia; EGL: external granular layer; GC: Golgi cells; IGL: internal granular layer; ML: molecular layer; PCL: Purkinje cell layer; SC: Stellate cells. (**B**) PCs were effectively captured at independent developmental stages. Scatter plot of the first two principal components (PC1/2) for all samples, displaying significant groupings according to postnatal day. 95% confidence ellipses for these groups are shaded in grey, displaying full separation. (**C**) Predominant capture of PC-specific transcripts. Gene expression data (RPKM values) across developmental time points (X-axis) for two cell-specific markers for each cell-type. CGN: cerebellar granule neurons; B/SC: basket/stellate cells; GC: Golgi cells; BG: Bergmann glia. See also Supplementary Fig. S1.

We set out to survey the intrinsic genetic program that drives PC development over this highly sensitive and critical period and to correlate this with the expression of ASD genes. To do so, we analyzed PC transcriptome dynamics in the developing mouse cerebellum during the first three postnatal weeks, a key developmental period that is equivalent to the third trimester in human cerebellar development^15^. PCs undergo extensive developmental changes during this period, including elaborate dendritic outgrowth and synaptogenesis (Fig. 1A)^16,17^.

PCs constitute considerably less than 1% of the total cerebellar cell population^18^. To identify PC-specific transcriptome dynamics that might otherwise be masked using a whole tissue approach, we employed laser capture microdissection to isolate individual PCs. Deep sequencing was performed on RNA isolated from 1000 PCs at five developmental time points (postnatal days P0, P4, P8, P14 and P21) in triplicate. Importantly, time points were separated in a principal component analysis (PCA) (Fig. 1B), demonstrating that PCs were effectively captured at independent developmental stages.

We next confirmed that PCs were isolated successfully and contributed the majority of the captured RNA. To do so, we compared the transcript abundance (RPKM values) of genes considered to be markers of PCs or other cerebellar cell populations, including cerebellar granule neurons, Basket and Stellate cells, Golgi cells and glia. The captured cells showed very high enrichment for PCs, with the PC-specific markers *Calb1* and *Itpr1* displaying strong expression that increased over postnatal development (Fig. 1C; Supplementary Fig. S1B). Markers for other cerebellar cell types demonstrated little-to-no expression. We nevertheless identified minor expression of a glial cell marker (*Gdf10*), suggesting a potential contamination by this cell type, likely due to the intimate physical association of Bergmann glia with PCs^19^. Together, these results demonstrate the successful isolation of a highly enriched population of PCs and the capture of gene expression in PCs across all time points.

**Figure 2:**
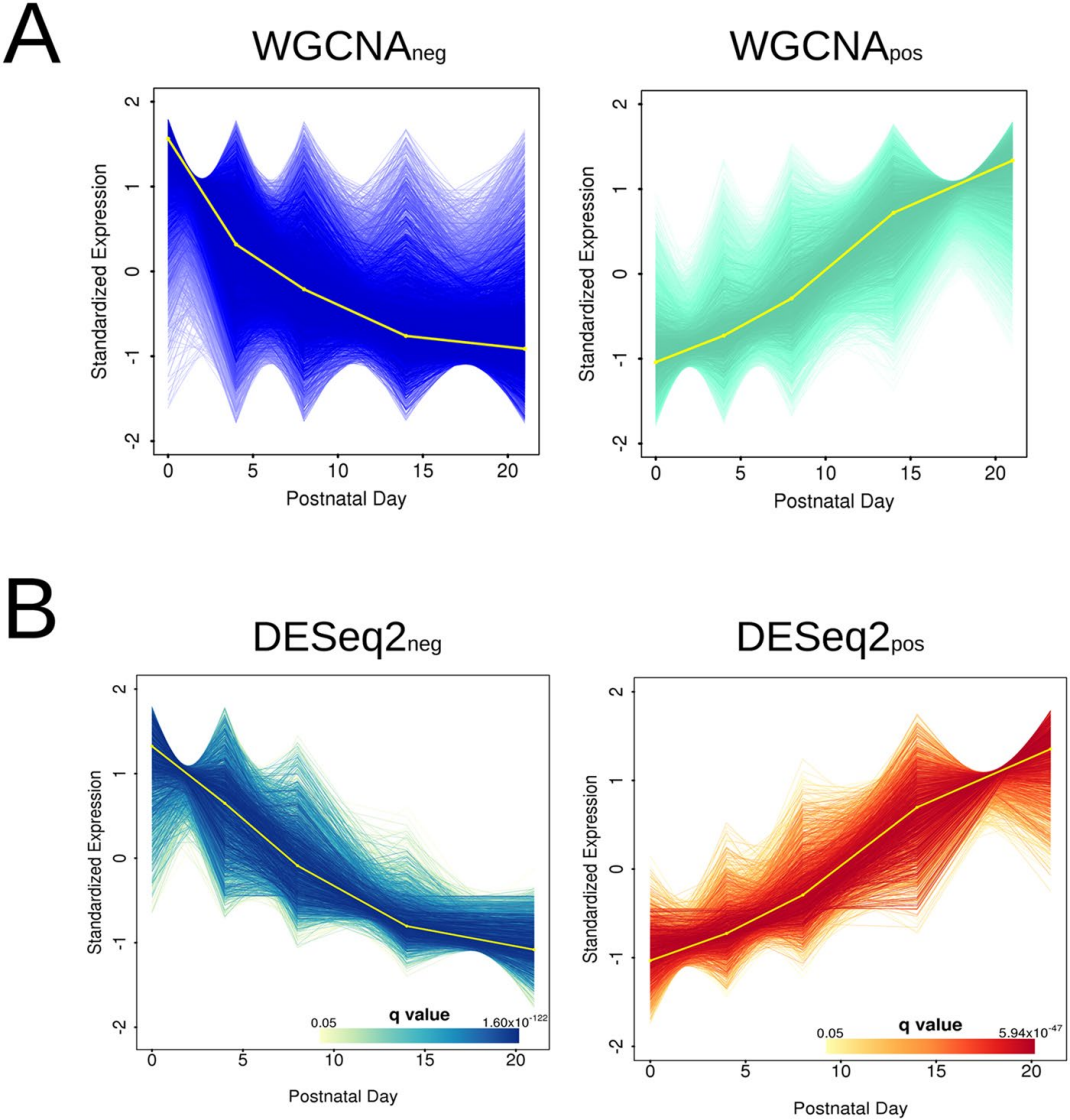
Co-expression network analysis reveals temporal gene clusters that are correlated with PC development. (**A**) Genes assigned to the two WGCNA modules with statistically significant functional annotations (q < 0.05), expressing patterns of negative (WGCNA_neg_) and positive (WGCNA_pos_) fold-change over time, respectively. WGCNA_neg_ is significantly enriched for ontology terms associated with RNA processing, while WGCNA_pos_ is significantly enriched for ontology terms associated with neuron formation and neurological disorders. See also Supplementary Figs S2 and S3. Expression values are normalized and standardized to a mean of zero and standard deviation of 1. The eigengene of the cluster is displayed in yellow. (**B**) Statistically significant differentially expressed genes over time, separated in clusters of negative (DESeq 2_neg_) and positive (DESeq 2_pos_) fold-change, respectively. The DESeq 2 clusters show strong intersection with those identified by WGCNA and provide robustness for downstream analyses. Both WGCNA and DESeq 2 clusters contained similarly enriched ontology terms. See also Supplementary Fige S4 and Supplementary Table S3. Each gene within the module is plotted as a line of increasing colour intensity with decreasing q value, as displayed in the colour scale. Expression values are normalized and standardized to a mean of zero and standard deviation of 1. The eigengene of the cluster is displayed in yellow.

We next investigated gene expression patterning across development using weighted gene co-expression network analysis (WGCNA)^20^. We defined 17 gene clusters (Supplementary Fig. S2) and then investigated each of these modules for both functional terms and disease-associated gene enrichment. Only two clusters (the aquamarine and the blue module) yielded significant functional annotation enrichments (Fig. 2A). The first cluster (WGCNA_neg_) contained 5,052 genes (680 with module membership >0.8) that were negatively correlated with PC maturation, and was significantly enriched for genes involved in RNA processing (Supplementary Fig. S3). The second cluster (WGCNA_pos_) was composed of 4,226 genes (1,084 with module membership >0.8) whose expression positively correlates with PC maturation. This cluster was significantly enriched in terms associated with neuronal development. Specifically, WGCNA_pos_ was enriched in genes whose disruption leads to long-term potentiation phenotypes, and abnormal nervous system electrophysiology and PC morphology (Supplementary Fig. S3). These findings are consistent with an enrichment of genes critical for postnatal PC development. Interestingly, abnormal PC development and early PC dysfunction are emerging mechanisms underlying many cerebellar ataxias^21,22^. Our data support this as we found a strong enrichment for the KEGG ataxia pathway in the WGCNA_pos_ gene cluster (Supplementary Fig. S3). Overall, these results confirm ontologies and pathways expected in PC development and suggest that we can exploit these data to explore the genetic correlates of ASD in developing PCs.

As the relatively low number of replicates per time point may affect the robustness of WGCNA clusters, we performed the same analyses using DESeq 2^23^ on genes that were identified as being significantly differentially expressed across time and correlated with PC maturation. We identified 2,511 and 1,917 significantly differentially expressed genes across time that were negatively (DESeq 2_neg_) and positively correlated (DESeq 2_pos_) with PC maturation, respectively (Supplementary Fig. S4). The WGCNA and DESeq 2 approaches were largely concordant: 82.5% and 97.0% of the genes within the negatively and positively regulated WGCNA clusters (module membership >0.8) were also found within the two DESeq 2 clusters, and gene set enrichments were similar (Supplementary Figs S3 and S4). We will refer to the positively correlated gene clusters WGCNA_pos_ and DESeq 2_pos_ as “PC development clusters”.

**Figure 3:**
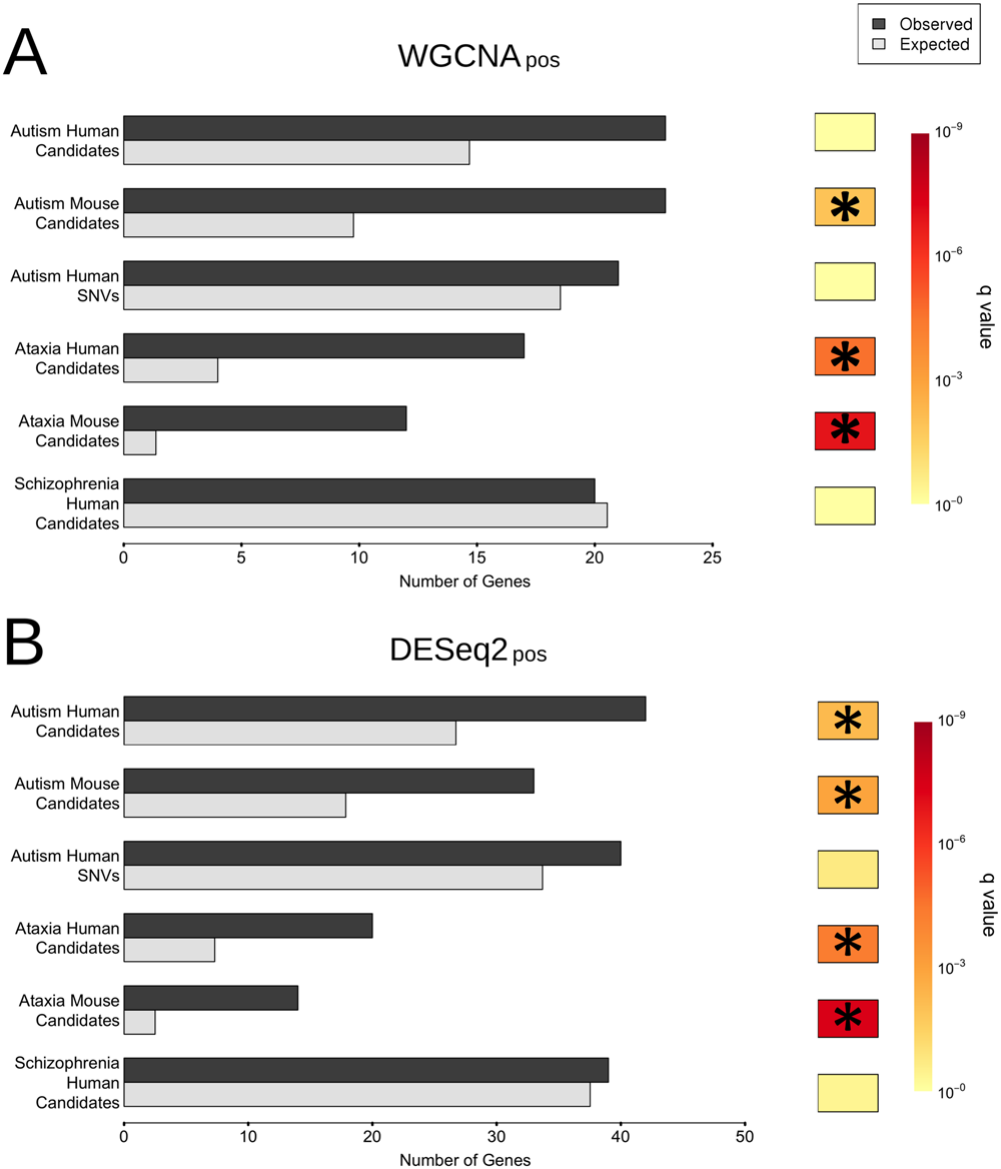
The developmental PC cluster positively correlated with timing contains unexpectedly high numbers of ataxia- or autism- associated genes. (**A**) Observed and randomly expected numbers of genes present in the WGCNA_pos_ PC cluster (module membership >0.8), for genes and SNVs associated with ataxia, autism, and schizophrenia (including both mouse model and human candidate lists for the former two disorders). q-Values are indicated using a Hinton plot (right), with significant results (*q* < 0.05) marked by an asterisk. See also Supplementary Table S4. (**B**) Analogous testing within the corresponding DESeq 2_pos_ cluster. This returned similar results, with significant enrichments for both autism gene lists (human and mouse, respectively). q-Values are indicated using a Hinton plot (right), with significant results (*q* < 0.05) marked by an asterisk.

Given the association of the cerebellum with various neurological diseases including ataxia but also cognitive disorders such as ASD^24^, we next investigated whether genes associated with these disorders were enriched in any of the identified gene clusters. Of the 17 WGCNA clusters, only the WGCNA_pos_ cluster returned significant enrichments for human genes causing ataxia (4.3-fold, *q* = 2.27 × 10^−5^) as well as genes that when disrupted in mice cause ataxia-like phenotypes (8.8-fold, *q* = 1.19 × 10^−7^) (Fig. 3A, Supplementary Table S4). The difference in enrichment observed between human and mouse likely stems from the fact that many genes in mouse do not have a one-to-one orthologous gene in human leading to a reduction in power.

We next interrogated the gene clusters for an enrichment for ASD-associated genes from the Simons Foundation Autism Research Initiative (SFARI) database^25^. The SFARI human database contains more than 800 human genes associated with ASD, while the mouse ASD-associated gene database contains a subset of these (229), for which genetic models have been generated. Interestingly, the WGCNA_pos_ cluster was the only cluster with a significant enrichment, for which we observed a 2.4-fold enrichment (*q* = 1.10 × 10^−2^) for mouse ASD-associated genes (Fig. 3A; Supplementary Table S5). Similar conclusions were reached for the cluster identified with DESeq 2 (Fig. 3B; Supplementary Table S5). Importantly, we also found a significant enrichment of orthologues for human genes associated with ASD within the DESeq 2_pos_ cluster (1.6-fold enriched, *q* = 6.34 × 10^−3^). We also used a recently published set of *de novo* variants associated with autism^26^ (Fig. 3B; Supplementary Table S5). We found no significant enrichment for these variants after multiple test correction in the clusters identified with WGCNA and with DESeq 2 (Fig. 3).

The difference between the WGCNA and DESeq 2 clusters likely stems from the greater number of genes within the latter, resulting in its greater analytical power. There was no significant enrichment for genes associated with schizophrenia in either cluster, suggesting that the observed enrichment is specific to ASD but not another related neurological disorder, namely schizophrenia (Fig. 3).

In addition to the ASD-associated genes from the SFARI database, we also investigated 842 genes whose transcripts were previously identified as targets of the Fragile X mental retardation protein (FMRP)^27^ and significantly enriched for ASD-associated genes^28,29^. FMRP targets were only enriched in the WGCNA_pos_ cluster (2.7-fold enrichment, *q* = 7.34 × 10^−21^) and both DESeq 2_pos_ and DESeq 2_neg_ clusters (2.3-fold and 1.4-fold enrichments; *q* = 2.78 × 10^−30^ and 1.02 × 10^−6^). These findings provide evidence that FMRP targets are enriched in developemtnally regulated PC genes.

**Figure 4:**
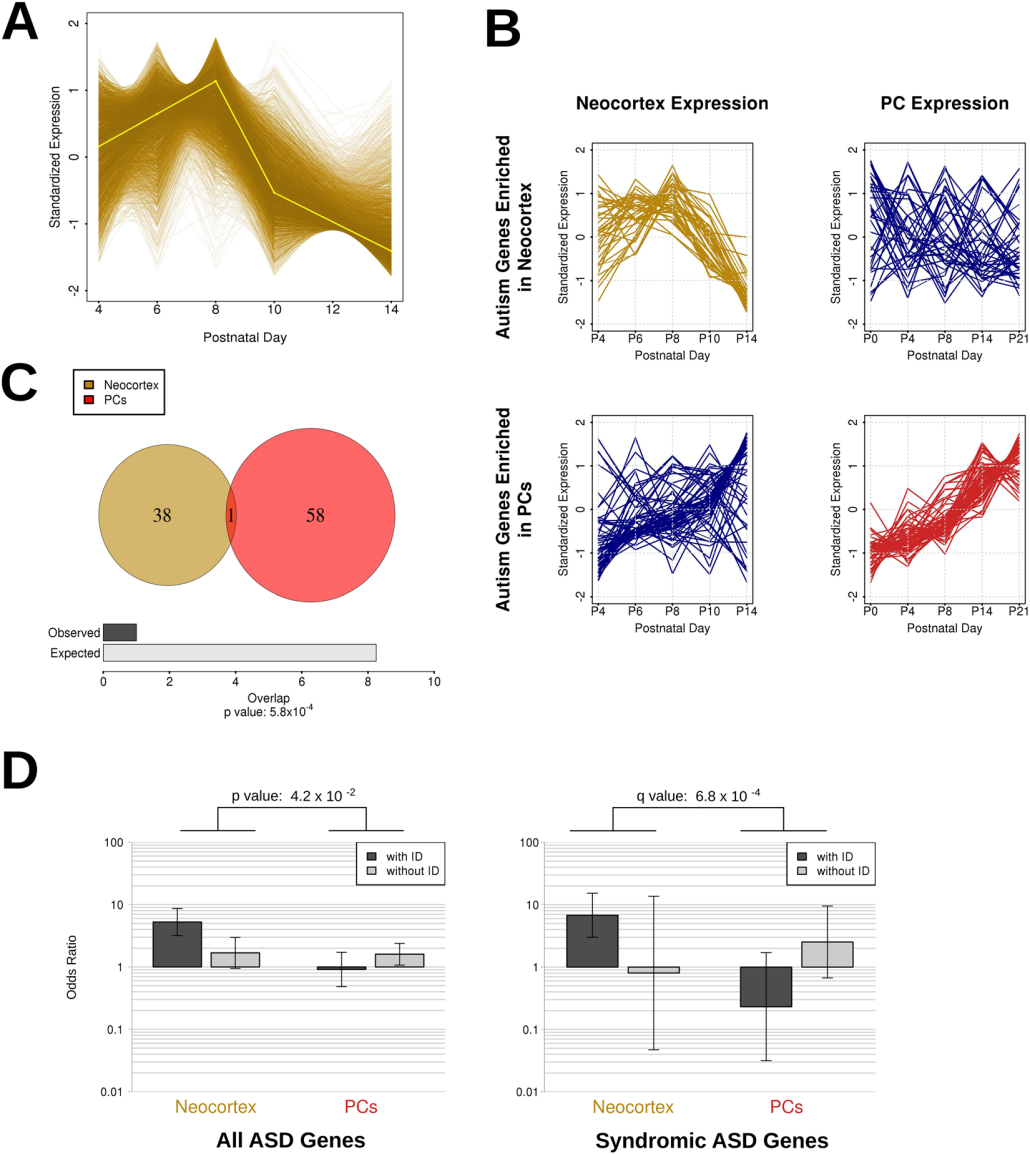
Comparisons between PC ASD (DESeq 2_pos_) and neocortex ASD gene clusters reveal differential ID co-morbidity. (**A**) The neocortex cluster exhibits statistically significant enrichment for autism genes (for either mouse models or human candidates – see also Supplementary Table S5). Each gene within the module is plotted as a line whose colour intensity is determined by module membership. Expression values are normalized and standardized to a mean of zero and standard deviation of 1. The eigengene is displayed in yellow. (**B**) The expression pattern of the autism-associated genes over development in either the neocortex or PCs shows a lack of coherency of each of the two modules for expression in the other tissue. Each gene within the module is plotted as a line, with expression values normalized and standardized to a mean of zero and standard deviation of 1. (**C**) Significant lack of overlap between the 182 autism-associated genes within the neocortex and PC clusters (Fisher’s exact test). (**D**) Intellectual disability (ID)-associated autism genes are significantly differently partitioned between the PC and neocortex gene clusters. Two sets of odds ratios from four contingency tables, including ID-associated and ID-free autism gene lists and their occurrences in both the neocortex cluster and the PC cluster. The two panels represent testing on autism gene lists for all ASD genes (syndromic genes and category 1–4 genes) and syndromic genes only. The p- and q-values indicate the estimated probability of the difference between the ratios of odds ratios for the two clusters.

We next investigated the specificity of these results with regards to PC development by undertaking an equivalent analysis on published transcriptomes from the developing mouse neocortex at the same postnatal time period^30^. For this analysis we limited our investigations to the sequencing libraries from layer 4, as it was the only layer fully isolated from other cortical layers^30^. Twelve WGCNA gene clusters were defined from these data (Supplementary Fig. S5), of which only one (the darkgoldenrod module) was significantly enriched for both mouse and human SFARI ASD genes (categories 1-4 and syndromic) (3.0-fold and 2.9-fold enrichments; *q* = 0.018 and 3.61 × 10^−4^, for the mouse and human gene sets, respectively; Supplementary Fig. S6). The enrichment analyses performed on category 1 or 2 genes did not yield significant results likely due to loss of statistical power associated with the low number of genes involved. In contrast to the gene clusters identified in developing PCs, this ASD-enriched neocortex cluster does not show positive differential expression over development (Fig. 4A). When using the variants identified by Krumm *et al*.^26^ we observed a low p-value (*p* = 0.029), but the enrichment was no longer significant after multiple test correction (*q* > 0.05). We identified a significant enrichment of FMRP target genes in two clusters. These included the ASD-enriched neocortex cluster (3.7-fold enrichment, *q* = 3.12 × 10^−8^) and a cluster positively correlated with developmental timing (1.85-fold enrichment, *q* = 3.96 × 10^−19^). Interestingly, we found that ASD-associated genes in the PC and neocortex clusters showed distinct spatiotemporal expression levels. Genes in the neocortical gene cluster exhibited low coherency and preservation over PC development, and *vice versa* (Fig. 4B). These results indicate that the two clusters are distinct and not maintained across development in different brain regions.

To test the degree of overlap between PC and neocortex cluster genes, we performed an intersection analysis of the ASD-associated genes within each identified cluster. Of 182 genes that occur in either the human or mouse ASD gene lists, 39 were present in the neocortex cluster and 59 occurred in the PC cluster (Fig. 4C). We found only a single gene (*Maoa*) that was present in both sets, which represents a statistically significant depletion (*p* = 5.8 × 10^−4^; Fisher’s exact test). The unexpectedly low overlap suggests that the observed enrichment of ASD genes in developing PCs is specific to this neuronal subpopulation at the investigated developmental time period.

The identification of the two spatiotemporally distinct ASD gene clusters in PCs and neocortex, respectively (Fig. 4B,C), allowed us to ask the intriguing question of whether genes within these cell type-specific modules might contribute to distinct ASD endophenotypes^31^. A stratification of the broad autism phenotype into clinically, genetically and biologically meaningful categories has thus far been lacking. One of the ASD endophenotypes that has been proposed to define clinical subgroups is intelligence quotient^32–34^. We therefore considered whether the two different gene clusters reflect differences in intellectual disability (ID) among ASD individuals. To do so, using the SFARI annotations, we split the human candidate ASD gene list into those associated with ID and those with no evidence for ID, and tested for their enrichment within either the PC or neocortex modules. Comparison of the odds ratios from these four contingency tables revealed that the PC gene cluster contains a significantly greater proportion of ID-free ASD-associated genes than the neocortex gene cluster. In contrast, this pattern is inverted in the neocortex cluster, with it containing a greater proportion of ID-associated ASD genes (*p* = 4.2 × 10^−2^; permutation test; Fig. 4D). By repeating this analysis on the syndromic SFARI genes separately, this inversion of patterns was found to be significantly pronounced (*q* = 6.8 × 10^−4^; permutation test; Fig. 4D) and the trend was maintained, albeit at a non-significant level, for categories 1-4 (*q* = 2.4 × 10^−1^; permutation test; data not shown). Of the 30 syndromic genes associated with ID, 8 were identified in the neocortex cluster, and only one in the PC cluster. Conversely, of the 11 syndromic genes with no reported association with ID, none were found in the neocortex cluster and 3 were found in the PC cluster. These results indicate that there is a significant difference in the composition of the ASD genes underlying the clusters in the two brain regions with relation to their association with ID. These differences suggest the existence of genetically and spatiotemporally distinct endophenotypes of ASD.

## Discussion

The analysis of gene expression within disease-relevant tissues and cell types is a powerful approach to identify genes and regulatory networks whose disruption is associated with disease. Given the wide heterogeneity of the ASD phenotype, it is expected that multiple brain regions, cell types and critical time periods contribute to the disease. Previous analyses indicated that ASD-associated genes expressed in the prefrontal cortex show a significant association with developmental patterning^29,35^. However, little attention has been paid to the ASD gene expression profiles in the cerebellum, a key brain region in ASD. Here, we report gene expression changes across key postnatal developmental stages within a population of laser-captured cerebellar cells that are highly enriched for Purkinje cells. Interestingly, we have identified a cluster of ASD genes that is enriched among all genes whose expression significantly increases over PC development. Importantly, the ASD genes enriched in the developing PC cluster are associated with a distinct disease endophenotype, namely the absence of ID co-morbidity. This is in contrast with a single, independent gene cluster in the mouse neocortex at the same developmental time point that is enriched for ASD genes with ID association. To our knowledge, these findings are the first to indicate a relationship between the spatiotemporal expression pattern of ASD genes and genetically distinct ASD endophenotypes.

A potential limitation of our study might stem from potential differences in developmental processes and developmental timing in the neocortex and the cerebellum owing to, for instance, neurogenesis occurring earlier in the neocortex. Such differences might partly explain the difference in the correlation between gene expression and development for the ASD gene-enriched neocortex and PC cluster, respectively. Furthermore, differential developmental gene expression might explain why some of the identified transcripts appear only in the PC cluster or neocortex cluster, despite their known widespread expression patterns.

Our identification of an ASD-gene enriched expression cluster that is specific to developing PCs provides support for the emerging concept that this neuron population is highly vulnerable in ASD^1–3,10^. Moreover, our findings inform future functional studies of the identified specific ASD candidate genes that should be carried out in developing PCs to obtain a deeper understanding of the pathophysiological mechanisms underlying ASD. It is important to note that we found some evidence of glial markers in our samples and thus cannot rule out the presence of some contaminating glia cells, most likely Bergmann glia that are present in the PC layer. However, given the very high abundance of PC markers compared to glial markers, the effect of Bergmann glia should be marginal and does not affect our conclusions that the developing cerebellum has an ASD signature. Other transcriptomic studies have identified cerebellar gene networks enriched in ASD genes that show partial overlap with our data. A correlation analysis of ASD genes with images from the Allen Mouse Brain Atlas^36^ found two co-expression modules that were significantly overexpressed in the cerebellar cortex including *Aldh5a1*, *Astn2*, *Auts2*, *Dpp10* and *Sez6l2*, which are also present in our developing PC gene cluster. Interestingly, a recent transcriptomic analysis in human post-mortem cerebellum from ASD and control individuals identified three co-expression modules significantly associated with ASD that include several genes present in the PC development cluster identified in our study, including *NDUFA5* as a hub gene in one of the downregulated clusters^37^. In addition, co-expression network analysis with human BrainSpan data identified an ASD gene enrichment including *ANK2* in thalamus and cerebellum during postnatal human development^38^. These studies support our findings but also suggest that gene networks in cerebellar cell populations other than PCs and across different developmental epochs are relevant to ASD. In future studies, it will be important to employ a similar approach to ours to all other subpopulations of the cerebellum and other brain regions across the entire developmental period in mouse but also human tissue. Advances in single-cell sequencing experiments across development are poised to identify and compare cell- and stage-specific expression signatures.

The identification of spatiotemporally distinct ASD gene clusters in the developing cerebellum and cortex that correlate with ID suggest genetically distinct ASD endophenotypes. These results raise the interesting prospect of genes and pathways associated with these modules being of use in predicting, upon initial diagnosis, eventual ASD phenotype severity, patient stratification and, ultimately, targets for therapeutic interventions.

## Methods

### Animals

Male C57/BL6 mice were used in this study. All animal work was approved by the University of Oxford Ethics Panel and in accordance with UK Home Office regulations.

### Laser Capture Microdissection and RNA Extraction

Laser capture microdissection was performed as previously described^39^. For the time points P4-P21, 1000 individual PC soma were collected randomly from 2–3 parasagittal tissue sections per mouse. For P0 cerebella, where it was difficult to isolate individual cells, clusters of PCs were isolated from 6 parasagittal tissue sections. Three biological replicates per time point were collected. Total RNA was extracted using the RNeasy Micro Kit (Qiagen) according to the Fibrous Tissues protocol. RNA quality was assessed on a 2100 BioAnalyzer using the RNA 6000 Pico Assay (Agilent Technologies). All RNA samples used for deep sequencing had an RNA Integrity Number (RIN) of ≥5. No principal components of the data were significantly correlated to the RIN values (q < 0.05).

### Library Preparation and Sequencing

cDNA libraries were prepared using the SMARTer® Ultra™ Low RNA Kit for Illumina® sequencing (Clontech), followed by NEBNext® DNA Library Prep Master Mix Set for Illumina® (New England Biolabs) according to manufacturers’ instructions except for the use of own custom indexes^40^. Sequencing of multiplexed, 100-bp paired-end libraries was done on an Illumina® HiSeq system using TruSeq SBS v3 chemistry. We acquired, on average, 61 million (range 38–100 million) paired-end 100-bp reads (Supplementary Table S2).

### Read Processing and Alignment

The same quality control and alignment procedures were followed for all RNA-sequencing data used in this study. Data quality was visualized through FastQC v0.9.2 (http://www.bioinformatics.babraham.ac.uk/projects/fastqc), and reads were trimmed using FASTX Toolkit v0.0.13 (http://hannonlab.cshl.edu/fastx_toolkit/index.html) and Cutadapt v1.2.1^41^ for adapter trimming and removal of other over-represented sequences. Reads were also trimmed when quality scores dropped below 20. For the 5′ paired-end Purkinje cell (PC) data and the single-end neocortex data, the reads were trimmed and retained, unless the resulting reads were less than 30 bp in length. Due to heavy adapter contamination in the 3′ paired-end PC data this was run in two iterations, firstly with more strict settings on the 3′ reads, followed by a second and less stringent run on the 5′ reads (seeking to maintain longer reads for guiding the alignment).

Alignment to the mouse reference genome GRCm38/mm10 was executed with the Genomic Short-read Nucleotide Alignment Program (GSNAP) of the Genomic Mapping and Alignment Program for mRNA and EST sequences (GMAP) package version 2012-07-20^42^. GSNAP was run for the paired-end PC data, with an estimated expected insert size of 220 bp and with an estimated deviation of 50 bp. GSNAP was run for the single-end read neocortex data with the same options (with no need for insert size parameters). Following this, for both datasets, only uniquely mapping reads were retained, and count tables were produced using HTSeq version 0.5.3p9^43^ with intersection-strict settings (a conservative measure only counting reads fully contained within features). All subsequent analyses in R were run within R version 3.1.1, and Bioconductor (biobase) version 2.26.0^44^.

Investigation into read quality returned evidence of substantial SMARTer adapter contamination^45^, from which initial trimming led to a mean loss of 4.89% of 5′ reads and 42.23% of 3′ reads. The contamination and resultant removal/orphaning of nearly half of all read pairs were most likely due to the low levels of RNA used for sequencing, with a mean concentration of 357 pg/μl and mean RIN value of 6.75. To circumvent this issue, heavier trimming was applied to the 3′ reads (to a minimum of 17 bp before discarding) while original thresholds were maintained for the 5′, therefore providing guidance during alignment with a specified estimated insert size of 220 bp (and allowed deviation of 50 bp), resulting in an average removal of 15.34% of the 3′ reads.

An equivalent procedure was followed for the neocortex data, using the data for the central layer (layer 4; the only layer completely isolated in this study). Please refer to Fertuzinhos *et al*.^30^ for more information on these neocortex libraries. The read trimming we applied based on quality scores resulted in a mean discarding of 0.31% of reads (0.26–0.38%), and read trimming due to contaminant removal resulted in a mean discarding of 3.75% of reads (2.58–6.35%). Of these remaining reads there was a mean of 98.38% (98.24–98.56%) of reads successfully mapped, with 59.95% (56.76–62.23%) having been uniquely mapped and retained for analysis.

Gene expression was assessed after read counting using HTSeq, and the normalization performed using DESeq 2^23^. RPKM were manually computed for each gene based on the number of read mapped. All analyses were performed on the normalized counts. Across all samples, a total of 20,928 expressed genes (coding and non-coding) with a minority of missing values across all samples were retained for analysis. We observed the mean correlation coefficients of gene expression values to be considerably higher between samples within time points (r^2^ = 0.93; range 0.86–0.98) than across time points (r^2^ = 0.65; range 0.34–0.95; Supplementary Fig. S1A). Picard metrics are reported in Supplementary Table S6.

### Weighted Gene Co-expression Network Analysis (WGCNA)

Using variance stabilized values from DESeq 2 we applied the WGCNA R package version 1.41.1^20^. Genes with greater than 50% missing values and/or zero variance were removed from analysis. Soft thresholding power value was set at 3 and 4 for the Purkinje cells and neocortex respectively, based on the scale-free topology fit (signed) and mean connectivity of the network. We set a minimum module size of 30 and the initial modules were merged based on eigengene identity using a dendrogram height of 0.5. Eigengenes were produced for each module by calculating their first principal components, thereby explaining the maximum amount of variation of expression levels. See Supplementary Table S7 and Supplementary Figs S2 and S5 for further details.

### GO, KEGG, and MGI and Disease Enrichment

All genes used in enrichment testing (gene-lists, clusters/modules, and backgrounds) were reduced to protein coding genes only. All enrichment testing was performed with Fisher’s exact tests providing q-values (Benjamini-Hochberg FDR corrected p-values), which were then corrected for gene-length bias using tools within the GOSeq R package version 1.18.0^46^. We used all genes with non-zero read count and non-zero variance within the Purkinje cells as a background gene set for the enrichment analyses. Gene Ontology enrichment was performed with GOSeq provided GO terms. GO terms were filtered through REVIGO (Reduce and VIsualize Gene Ontology)^47^, which labels terms exhibiting semantic similarity as redundant, with strict settings of allowed similarity at 0.5. The KEGG (Kyoto Encyclopedia of Genes and Genomes) database^48^ and MGI (Mouse Genome Informatics) database^49^ enrichment tests were performed with pathway and phenotype information accessed through KEGGREST version 1.6.0 and BioMART version 2.22.0, respectively.

For disease enrichment testing with human gene lists, these were translated to their corresponding orthologs in mice, and all counts were reduced further to those with 1:1 orthologs (between human and mice) only. Enrichment for ataxia-associated genes was tested with candidate lists obtained through literature searches (Supplementary Table S1). Testing with autism-associated genes used both human candidate and mouse model genes obtained from the SFARI database (accessed 2^nd^ Dec 2014)^25^. The SFARI resource annotates genes by degrees of confidence of their associations with ASD (1–6, decreasing in confidence), and by whether they are associated with syndromic forms of ASD. Only genes belonging to SFARI categories 4 to 1 (“minimal evidence”, “suggestive evidence”, “strong candidate” and “high confidence”) were used to conduct enrichment analyses. Testing with schizophrenia-associated genes used a human candidate gene list^50^. We used genes found to be expressed in the tissue of interest (PCs or neocortex) and with one-to-one orthologs in human and mouse as the background gene set.

### Differential Expression Analysis

Differential expression analysis was performed with the R package DESeq 2 version 1.6.2^23^. This provided a platform for applying a likelihood ratio test, suitable for time-series analysis with the aim of comparing models with and without time factor. Significant results were determined as those with a q-value < 0.05, and these were subsequently separated into positive and negative fold-change, providing two clusters. DESeq 2 statistics are reported in Supplemental Table S8.

### Permutation Testing

Permutation testing was performed to statistically quantify the differing trend in odds ratios with an empirical p-value. For each permutation, all four contingency tables and subsequent odds ratios were simulated (with matching sampling distribution) using Patefield’s algorithm, and those tables containing zeroes were adjusted with Haldane’s correction. From these, the log Ratio of Odds Ratio (log_10_ ROR) was calculated for each tissue, before taking the absolute difference between these. For each test, these tables were permuted 10^8^ times. The test was run twice on stratified candidate lists, once with syndromic ASD human candidates, and then with all other ASD human candidates. The p-values from the two stratified runs of permutation testing were adjusted for multiple testing, with significant results determined as those FDR < 0.05.

### Accession Codes

Sequencing data have been deposited into the GEO repository (accession number GSE86824).

## Supplementary information

Supplementary Information

Table S1

Table S2

Table S3

Table S4

Table S5

Table S6

Table S7

Table S8
