# Supplementary material for: A gene expression signature in developing Purkinje cells predicts autism and intellectual disability co-morbidity status
Source: Sci Rep. 2019 Jan 24;9:485. doi: 10.1038/s41598-018-37284-1 (PMC6346046; doi:10.1038/s41598-018-37284-1)
Supplement: Supplementary file 1 — Supplementary Information [file 41598_2018_37284_MOESM1_ESM.pdf]

Supplementary Information

A gene expression signature in developing Purkinje cells predicts autism and intellectual disability co-morbidity status

Harry Clifford[1], Anna Dulneva[1], Chris P. Ponting[1], Wilfried Haerty[1,2*] and Esther B. E. Becker[1*]

[1]MRC Functional Genomics Unit, Department of Physiology, Anatomy and Genetics, University of Oxford, Oxford, OX1 3PT, United Kingdom
[2]Current address: Earlham Institute, Norwich Research Park, Norwich, NR4 7UG, United Kingdom

*Corresponding authors: esther.becker@dpag.ox.ac.uk, wilfried.haerty@tgac.ac.uk

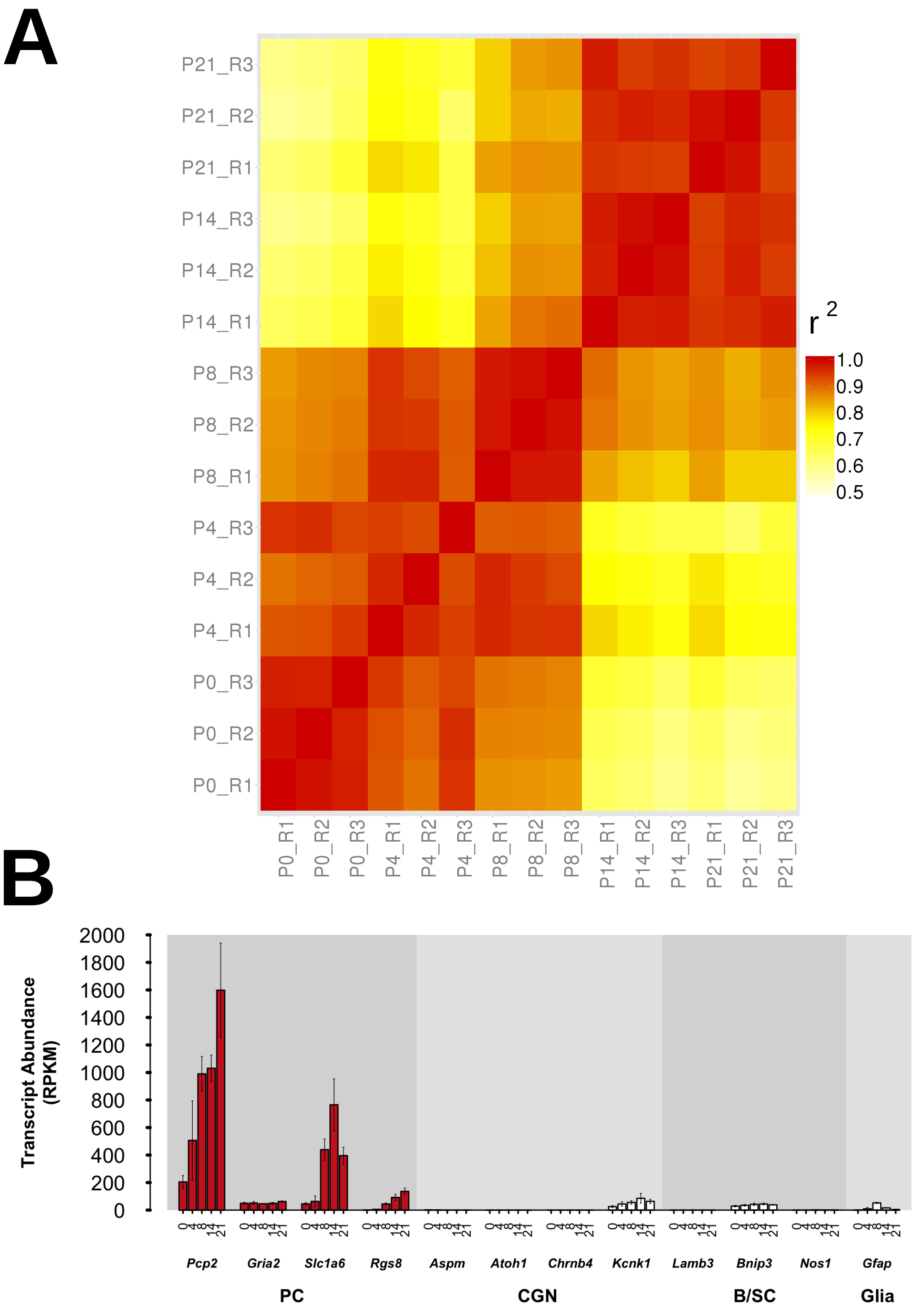

Supplementary Figure S1. Related to Figure 1 and Experimental Procedures. Gene expression correlations and for additional cell-specific markers confirms successful Purkinje cell capture.
(A) Heatmap of correlations (r^2) for gene expression data between samples. Overall, correlations are at their highest when comparing samples within a time point, and these decrease when comparing increasingly distant time points.
(B) Bar plots representing gene expression data (RPKM) across developmental time points for additional cell-specific markers, including Purkinje cells (PC), Cerebellar Granule Neurons (CGN), Basket/Stellate Cells (B/SC), and glia.

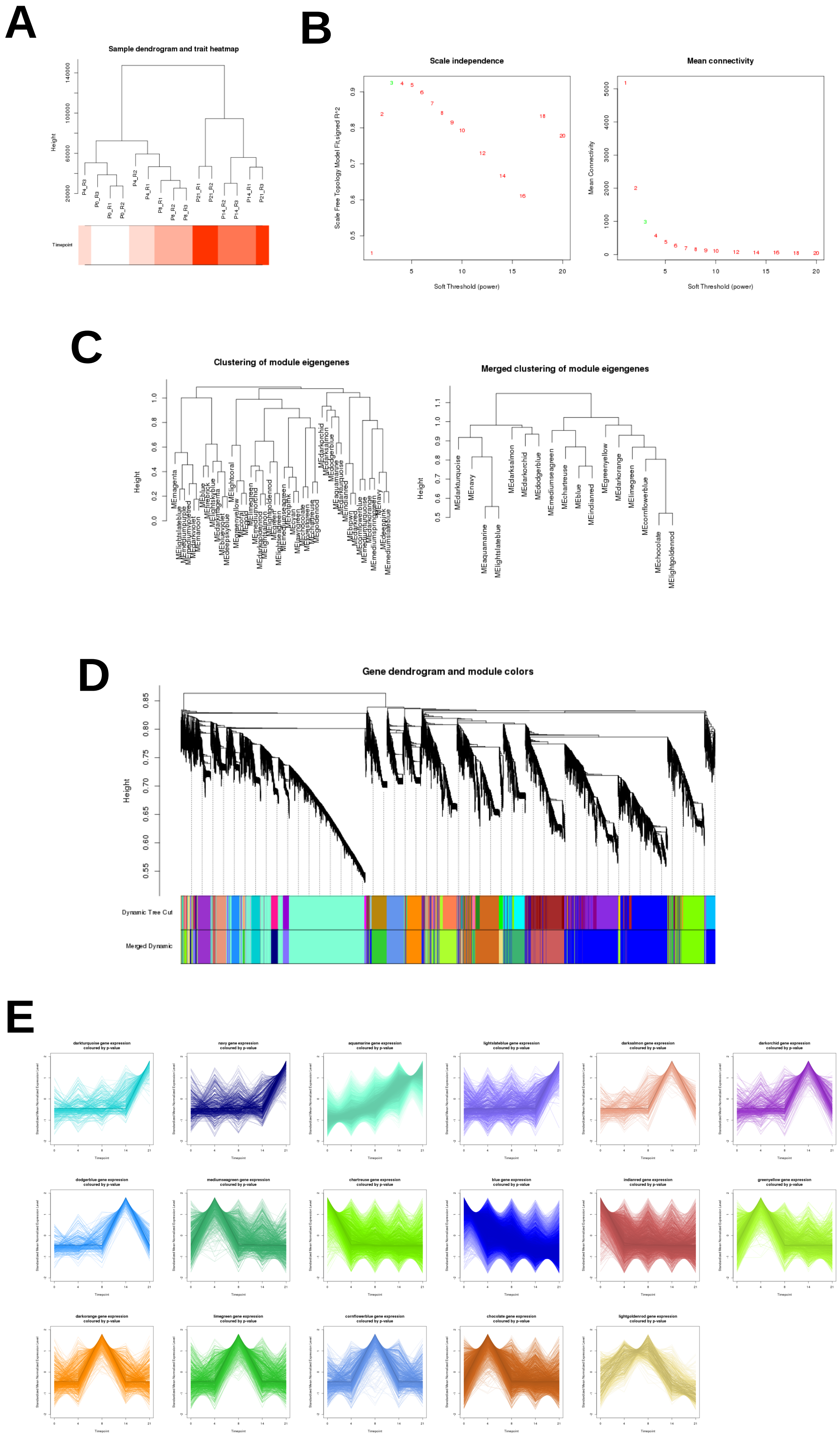
A
Sample dendrogram and trait heatmap
Height
Timepoint
B
Scale independence
Mean connectivity
Scale Free Topology Model Fit,signed R^2
Mean Connectivity
Soft Threshold (power)
C
Clustering of module eigengenes
Merged clustering of module eigengenes
Height
D
Gene dendrogram and module colors
Height
Dynamic Tree Cut
Merged Dynamic
E

Supplementary Figure S2. Related to Figure 2. WGCNA Analysis of Purkinje cell transcripts.

(A) Following the initial removal of genes with zero expression and/or zero variance, hierarchical clustering results (Euclidean distance and average linkage method) show no sample outliers.

(B) Soft thresholding power value was set at 3, based on the scale-free topology fit (signed) and mean connectivity of the network.

(C) Minimum module size was set at 30, with robust-sized modules ensured through merging of similar modules, determined as those whose eigengenes cluster (1-correlation distance and average linkage method) at a height of < 0.5.

(D,E) 17 final modules as displayed in the WGCNA gene tree plot and as a line chart of standardized expression over time point (one gene = one line). All modules were enrichment tested.

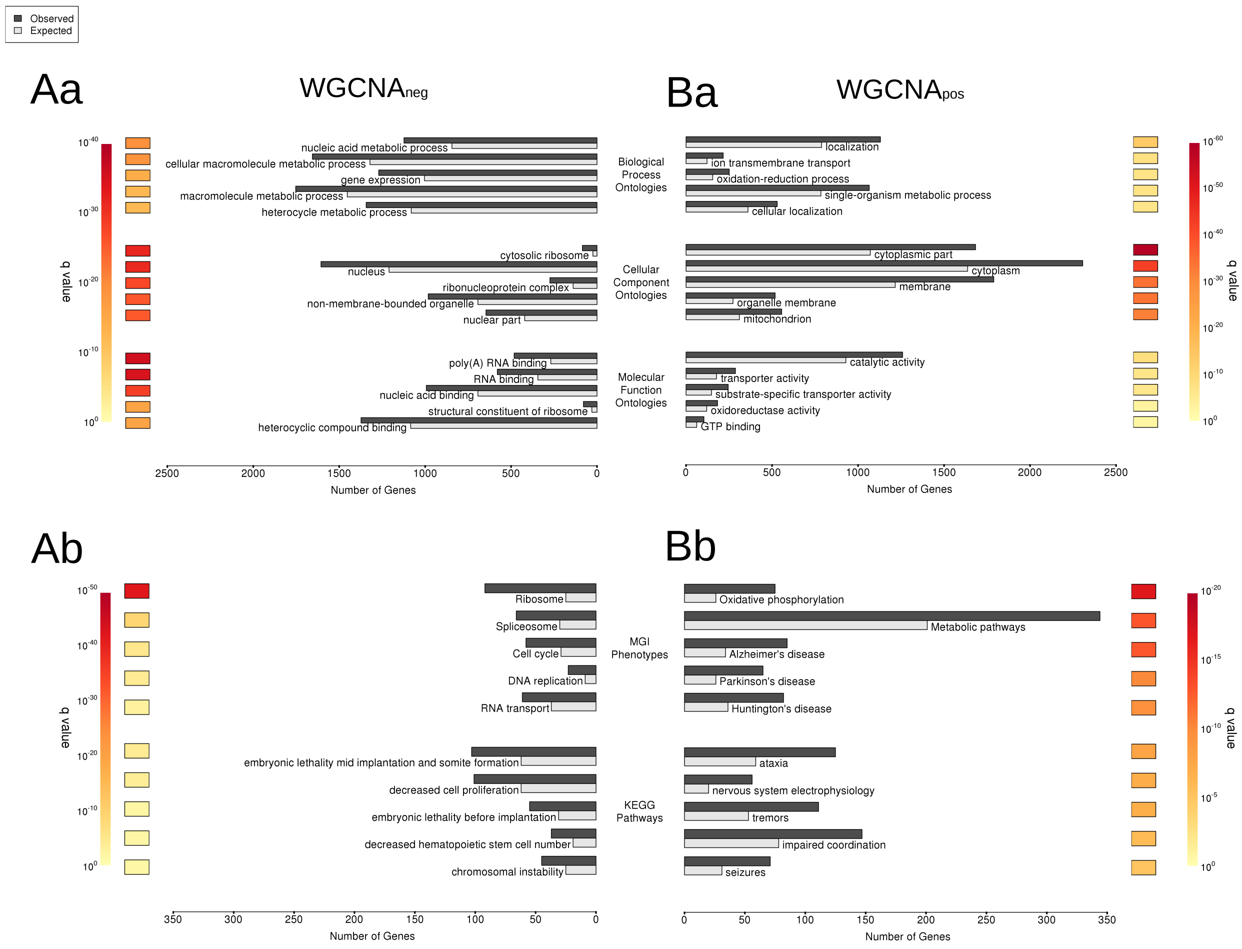

Supplementary Figure S3. Related to Figure 2. Functional annotation of WGCNA gene clusters.

Bar plots of the five most statistically significant enrichments (by p-value) for gene ontology terms (biological process, cellular component, molecular function) (top panels) and for phenotype (MGI) and pathway (KEGG) (bottom panels) within the respective WGCNA clusters. Bars represent the number of genes identified, allowing comparison of the observed number with the expected number. q-Values are indicated using Hinton plots. $WGCNA_{neg}$ displays associations of two clusters with RNA processing and $WGCNA_{pos}$ is significantly enriched in terms associated with neuron formation and neurological disorders.

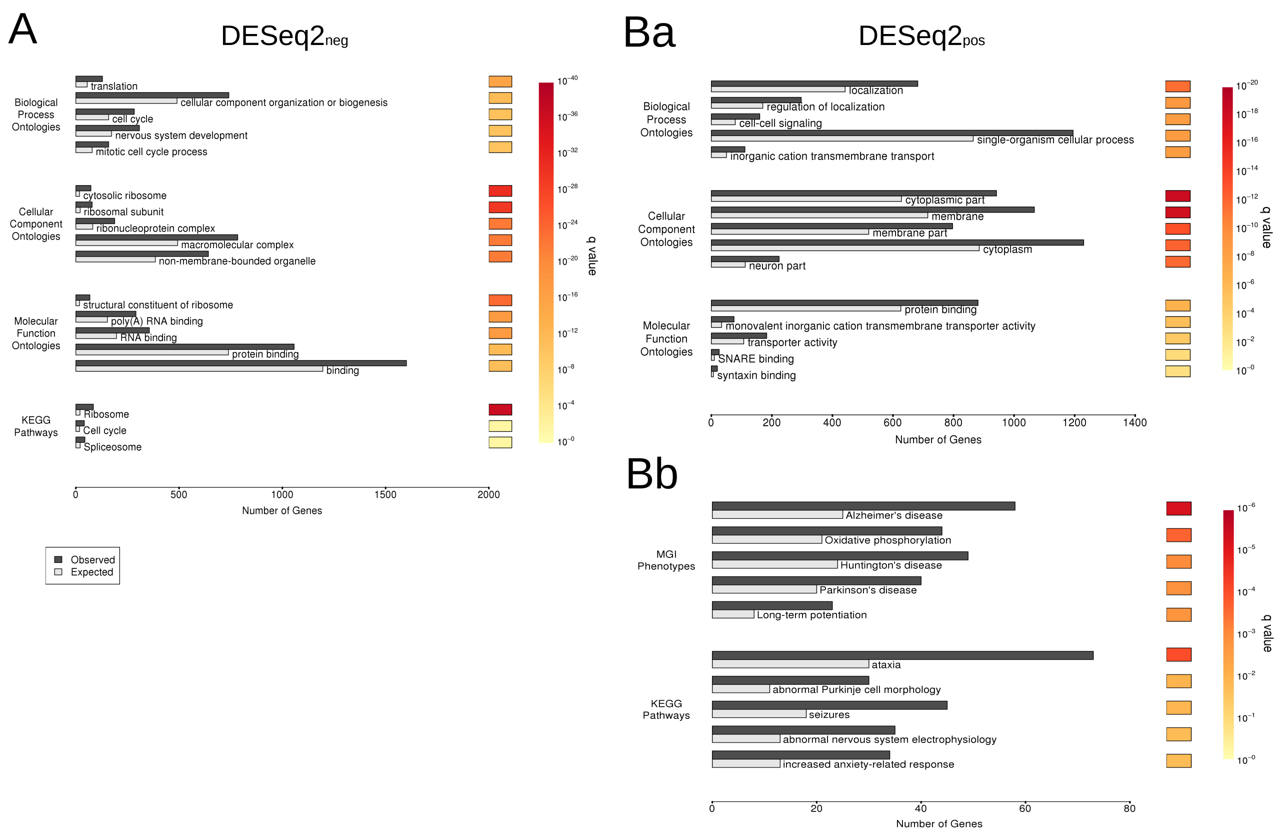

Supplementary Figure S4. Related to Figure 2. Functional annotation of PC clusters formed by differential expression analysis (DESeq2) displays similar ontology enrichments to the WGCNA analysis.

Bar plots of the five most statistically significant enrichments (by p-value) for gene ontology terms (biological process, cellular component, molecular function) (top panels) and for phenotype (MGI) and pathway (KEGG) (bottom panels) within the respective DESeq2 clusters. There are only three significant KEGG pathways and zero significant MGI phenotypes for the DESeq2$_{neg}$ cluster (left panels). All results presented are statistically significantly enriched. Bars represent the number of genes identified, allowing comparison of the observed number with the expected number. q-Values are indicated using Hinton plots.

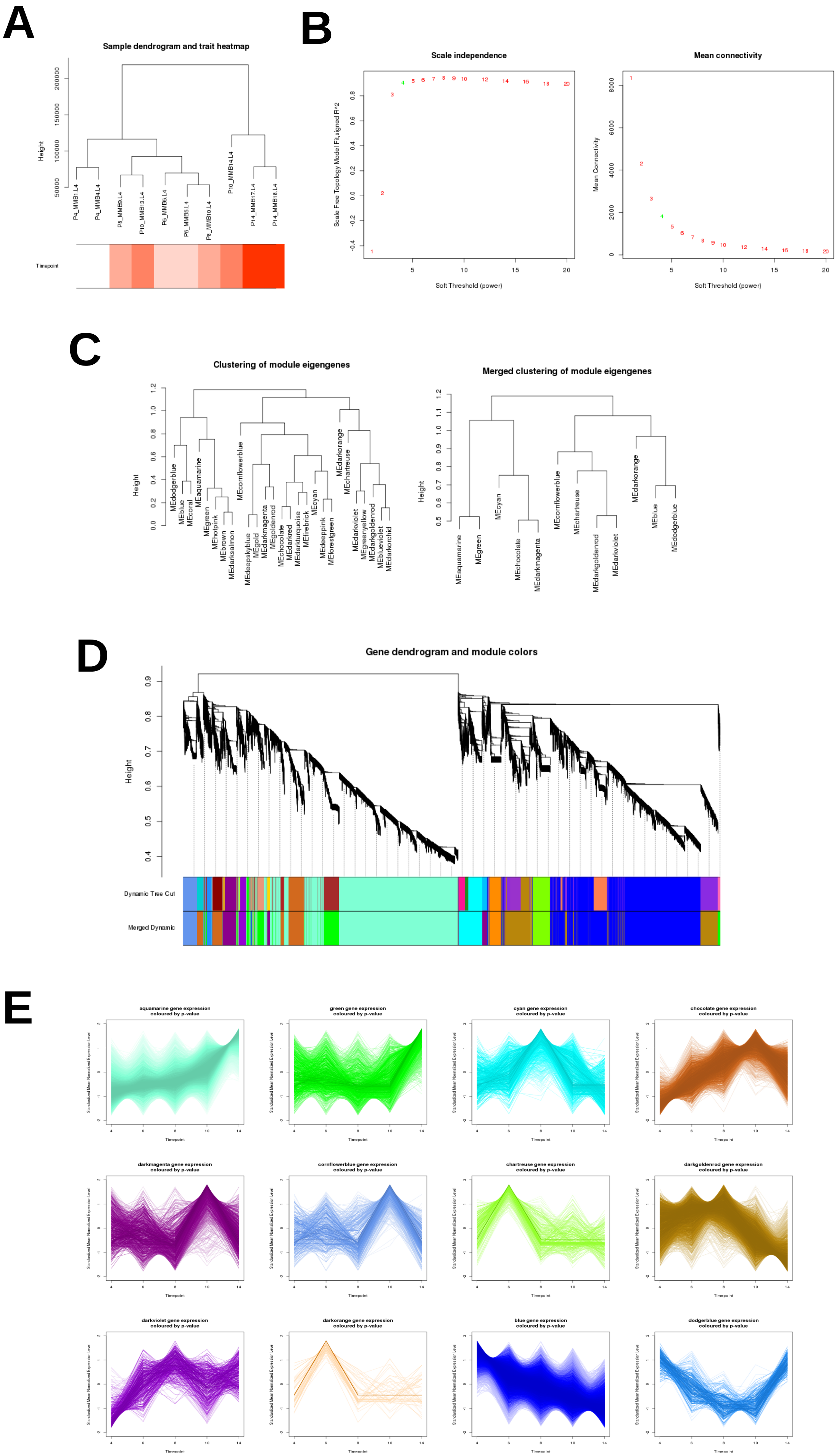
A
Sample dendrogram and trait heatmap
Height
P4_MMB1.L4
P4_MMB4.L4
P8_MMB9.L4
P10_MMB13.L4
P6_MMB6.L4
P6_MMB5.L4
P8_MMB10.L4
P10_MMB14.L4
P14_MMB17.L4
P14_MMB18.L4
Timepoint
B
Scale independence
Scale Free Topology Model Fit,signed R^2
Soft Threshold (power)
Mean connectivity
Mean Connectivity
Soft Threshold (power)
C
Clustering of module eigengenes
Height
Merged clustering of module eigengenes
Height
MEaquamarine
MEgreen
MEcyan
MEchocolate
MEdarkmagenta
MEcornflowerblue
MEchartreuse
MEdarkgoldenrod
MEdarkviolet
MEdarkorange
MEblue
MEdodgerblue
D
Gene dendrogram and module colors
Height
Dynamic Tree Cut
Merged Dynamic
E
aquamarine gene expression coloured by p-value
green gene expression coloured by p-value
cyan gene expression coloured by p-value
chocolate gene expression coloured by p-value
darkmagenta gene expression coloured by p-value
cornflowerblue gene expression coloured by p-value
chartreuse gene expression coloured by p-value
darkgoldenrod gene expression coloured by p-value
darkviolet gene expression coloured by p-value
darkorange gene expression coloured by p-value
blue gene expression coloured by p-value
dodgerblue gene expression coloured by p-value
Timepoint

Supplementary Figure S5. Related to Figure 4. WGCNA Analysis of neocortical transcripts.

(A) Following the initial removal of genes with zero expression and/or zero variance, hierarchical clustering results (Euclidean distance and average linkage method) show no sample outliers.

(B) Soft thresholding power value was set at 4, based on the scale-free topology fit (signed) and mean connectivity of the network.

(C) Minimum module size was set at 30, with robust-sized modules ensured through merging of similar modules, determined as those whose eigengenes cluster (1-correlation distance and average linkage method) at a height of < 0.5.

(D,E) 12 final modules as displayed in the WGCNA gene tree plot and as a line chart of standardized expression over time point (one gene = one line). All modules were enrichment tested.

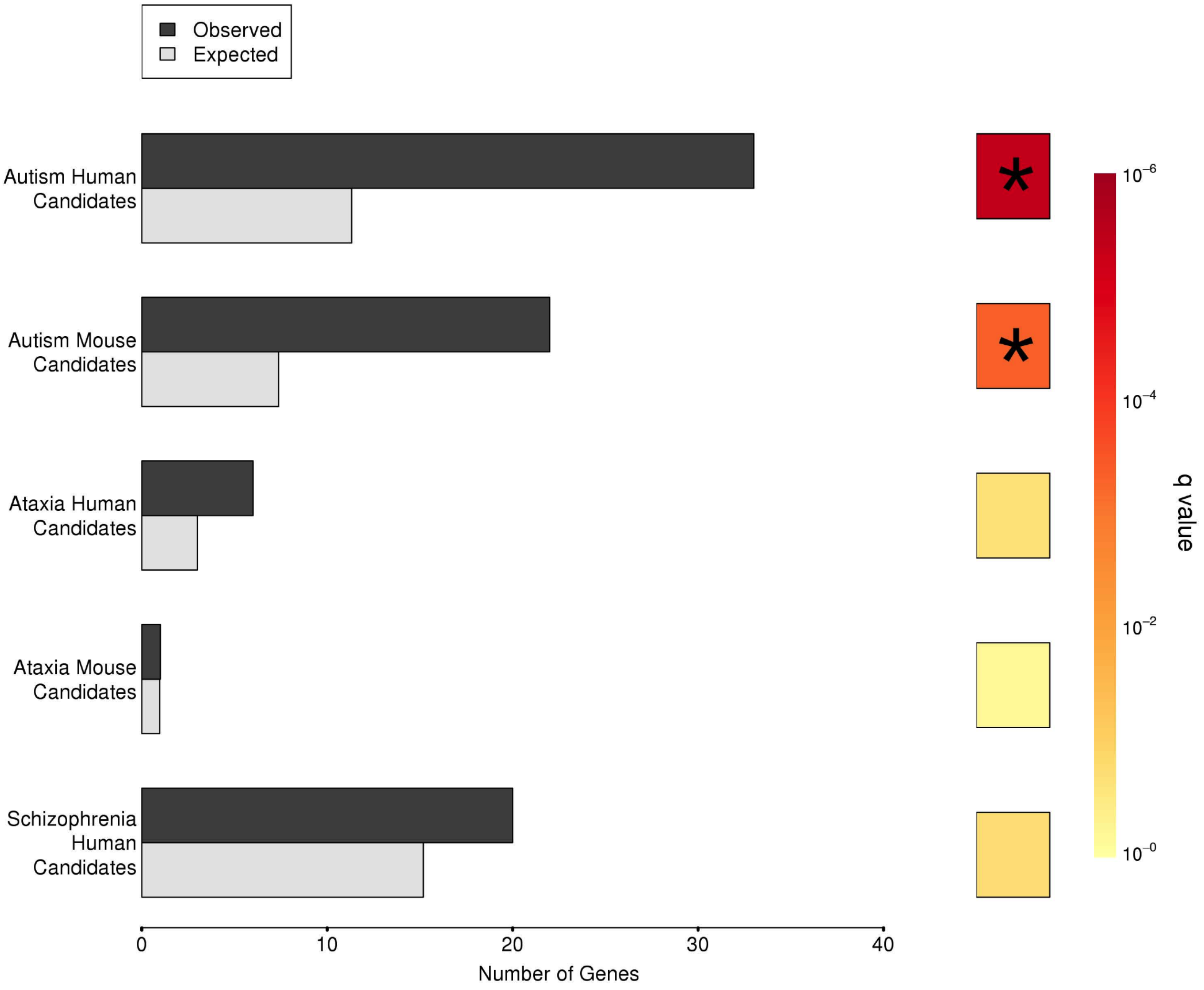

Supplementary Figure S6. Related to Figure 4. The neocortex cluster significantly enriched for autism-associated genes displays strong enrichment in both human candidate and mouse model gene lists.

Observed and randomly expected numbers of genes present in the neocortex autism-associated cluster, for genes associated with ataxia, autism, and schizophrenia. q-Values are indicated using a Hinton plot (right), with significant results ($q<0.05$) marked by an asterisk.

Supplementary Table Legends

Supplementary Table S1. Ataxia gene lists.
Ensembl IDs and common gene names for the curated ataxia-associated gene lists used during disease enrichment analyses.

Supplementary Table S2. Related to Methods. Readcounts.
Table including the number of reads sequences, and resultant alignment percentages.

Supplementary Table S3. Related to Figure 2. PC cluster: all GOs.
Full table of enrichment analyses for the Purkinje cell $DESeq2_{pos}$ cluster, including GO, MGI, and KEGG. This includes data on the number of genes identified, their relative enrichments, and subsequent q-values. This is an expansion of the data presented in Figure S3.

Supplementary Table S4. Related to Figures 3A and S2E. Enrichment testing. Enrichment testing data for disease-associations (as in Fig 3A) for all PC clusters (as in Fig S2E)

Supplementary Table S5. Related to Figure 3. Autism-associated cluster genes and *in situ* hybridization images.
Table of the autism-associated genes identified in the two tissue autism-associated clusters (neocortex and PC $DESeq2_{pos}$), and, where available, in-situ hybridization images from stages P4 and P14 of mouse development (Image Credit: Allen Institute. © 2008 Allen Institute for Brain Science. Allen Developing Mouse Brain Atlas. Available from: http://developingmouse.brain-map.org/).

Supplementary Table S6. Related to Methods. Picard Metrics.

Supplementary Table S7. Related to Methods. WCNA Statistics.

Supplementary Table S8. Related to Methods. DESeq2 Statistics.
